# Industrial Waste-Derived Carbon Materials as Advanced Electrodes for Supercapacitors

**DOI:** 10.3390/nano13222924

**Published:** 2023-11-09

**Authors:** Ge Bai, Wen Guo, Gang Wang, Bin Dai, Lu Liu, Lili Zhang, Feng Yu

**Affiliations:** 1 Key Laboratory for Green Processing of Chemical Engineering of Xinjiang Bingtuan, School of Chemistry and Chemical Engineering, Shihezi University, Shihezi 832003, China; baigemry700@163.com (G.B.);; 2 Institute of Sustainability for Chemicals, Energy and Environment, Agency for Science, Technology and Research, Jurong Island, Singapore 627833, Singapore; liu_lu@isce2.a-star.edu.sg; 3 Clean Energy Conversion and Storage Research Group, Bingtuan Industrial Technology Research Institute, Shihezi University, Shihezi 832003, China

**Keywords:** activated carbon, petroleum coke, dye wastewater, supercapacitor

## Abstract

Strategically upcycling industrial wastes such as petroleum coke and dye wastewater into value-added materials through scalable and economic processes is an effective way to simultaneously tackle energy and environmental issues. Doping carbon electrodes with heteroatoms proves effective in significantly enhancing electrochemical performance through alterations in electrode wettability and electrical conductivity. This work reports the use of dye wastewater as the sole dopant source to synthesize N and S co-doped petroleum coke-based activated carbon (NS-AC) by the one-step pyrolysis method. More importantly, our wastewater and petroleum coke-derived activated carbon produced on a large scale (20 kg/batch) shows a specific surface area of 2582 m^2^ g^−1^ and an energy density of about 95 Wh kg^−1^ in a soft-packaged full cell with 1 M TEATFB/PC as the electrolyte. The scalable production method, together with the green and sustainable process, can be easily adopted and scaled by industry without the need for complex processes and/or units, which offers a convenient and green route to produce functionalized carbons from wastes at a low cost.

## 1. Introduction

Energy and environmental sustainability are the challenges the world is facing, which stimulate tremendous research globally. Although there are renewable and clean energy sources such as wind and solar, they are largely limited by geographical features and land scale. Therefore, there is an urgent demand for efficient and cost-effective energy storage and conversion technologies [1,2,3], such as supercapacitors, batteries, and fuel cells [3,4,5,6,7,8,9]. A supercapacitor provides high power density and outstanding stability, but with relatively low energy density [10]. It plays an important role in next-generation energy storage systems for potential applications in ubiquitous portable electronics, power backups, hybrid vehicles, and other applications where pulse power or high power density is required [11].

Electrode material as an important part of supercapacitors is the focus of much research, and the commercial electrode materials for supercapacitors are mainly activated carbon (including graphene-like carbon [12,13], carbon nanotubes [14], porous carbon [15], etc.). Properties of the carbon, such as pore structure, heteroatom doping, etc., affect the performance of the electrode [16]. High specific surface area and abundant open pore structure can provide sufficient active sites for the adsorption and desorption of ions and facilitate electron transfer within the electrode. Heteroatom doping (N, S, O, etc.) can improve the conductivity and surface wettability of the electrode material while providing additional pseudocapacitance to enhance the specific capacitance [17,18]. Gan [16] reported that a porous carbon material with a large specific surface area doped with N/S derived from small organic molecules presents a specific capacitance of 280 F g^−1^. Wei [19] synthesized a nitrogen-doped ordered mesoporous carbon material using F127 as a template, showing a high specific capacitance of 288 F g^−1^ and good stability. The general doping strategy usually requires expensive N and S precursors, which increases the cost of the materials and limits industrial applications. Therefore, it is important to develop a strategy to synthesize inexpensive, high-performance functionalized carbon materials.

On the other hand, water pollution has caused widespread concern due to its wide range of influences and numerous sources of pollution [20]. More than 80% of the wastewater from human activities is discharged without purification. The textile industry consumes a huge amount of dye every year with low utilization and efficiency, resulting in a large quantity of dye wastewater, which is biologically toxic and difficult to degrade. The common method to treat dye wastewater is flocculation and adsorption [21]. However, both methods produce large amounts of sludge that are eventually landfilled or incinerated, which will cause secondary pollution. In view of these problems, the treatment of dye wastewater is a global challenge, and it is highly desirable to find an alternative and sustainable solution [13,22,23]. 

In this paper, we strategically tackle both problems, integrate them, and turn waste into valuables. Specifically, we use the dye wastewater, which contains rich azo and sulfite functional groups [24], as the sole N and S sources to synthesize heteroatom-doped petroleum coke-based activated carbon. By incorporating the dye wastewater into petroleum coke, we are able to make N and S co-doped carbon with a high specific surface area of 2582 m^2^ g^−1^ at a large scale of 20 kg/batch. When used as the electrode material, the heteroatom-doped carbon shows a specific capacitance of 234.4 F g^−1^ at 0.5 A g^−1^ with high energy density. This novel synthesis strategy does not only provide an alternative and sustainable method to treat industrial dye wastewater but also paves the path to upgrading petroleum coke, which is a by-product from the chemical industry, to functionalized activated carbon in an inexpensive and efficient way. The advantages of our proposed method include: (Ⅰ) simultaneous treatment, recovery, and utilization of the dye wastewater; (Ⅱ) obtaining heteroatom-doped activated carbon without additional nitrogen and sulfur sources; (Ⅲ) an industrially scalable and inexpensive process to upgrade petroleum coke by-products to high-value functionalized activated carbon; and (Ⅳ) the N,S co-doped activated carbon yields a large specific surface area and is suitable as electrode material and possibly for many other applications. Our proposed concept can also be extended to other fields, including but not limited to general wastewater treatment; air pollution control; and carbon capture, utilization, and storage.

## 2. Experimental

### 2.1. Preparation of Samples

**Figure 1:**
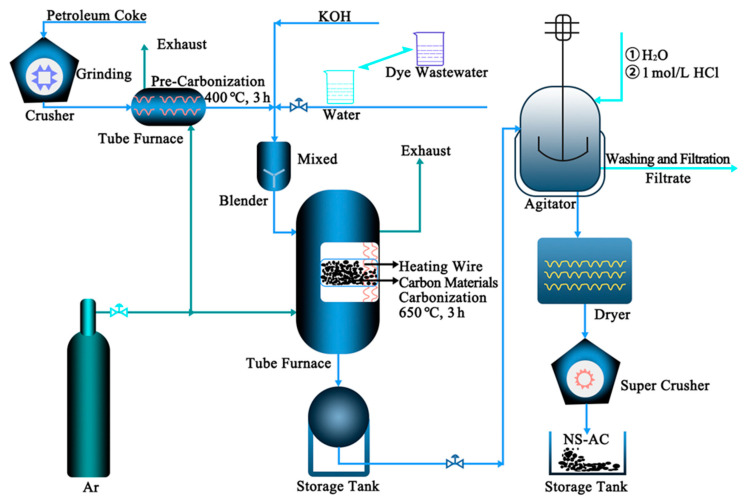
Schematic diagram for the upscale fabrication of AC.

The bulk petroleum coke was ground through a crusher and passed through an 80-mesh sieve. The industrial dye wastewater was spray dried to obtain a powdery solid for subsequent use. The petroleum coke (PC) powder was pretreated at 400 °C for 3 h with a heating rate of 5 °C min^−1^ in a tube furnace protected by Ar atmosphere. The petroleum coke powder, potassium hydroxide, and dye powder were dispersed in 10 mL of deionized water in a mass ratio of 2:5:0, 2:5:0.25, 2:5:0.5, and 2:5:1.0, respectively. The mixture was then dried, and the obtained slurry was placed in a corundum crucible in a tube furnace protected by an Ar atmosphere. The mixture was heated at 650 °C for 3 h at a heating rate of 5 °C min^−1^ to obtain bulk material. After washing with deionized water and 1 M HCl, the NS-AC (nitrogen–sulfur co-doped activated carbon) material was obtained after drying in an oven for 12 h. The resultant samples were labeled as AC, NS-AC-0.25, NS-AC-0.50, and NS-AC-1.00, respectively. PC was pristine carbon without activation. The schematic diagram of the upscale fabrication process is shown in Figure 1. The preparation process of NS-AC-0.5-S20 was the same, except that the amount of petroleum coke was 20 kg.

### 2.2. Characterization

Powder X-ray diffraction (XRD) patterns were recorded on a Bruker D8 Advance X-ray diffractometer (Bruker Biosciences Corporation, Billerica, MA, USA) with Cu Kα radiation (40 kV, 40 mA, λ = 1.5406 Å), with a scan rate of 10° min^−1^ at an angle of 10° to 90°. High-resolution transmission electron microscopy (HRTEM) images and EDS mapping were recorded on a FEI Tecnai G2 F20 (Standord, CA, USA) equipped with an energy-dispersive X-ray spectroscope (EDS, X-MAX80, JEOL, Taiwan), operating at 200 kV. N_2_ adsorption and desorption experiments were performed using an analyzer model from Micromeritics ASAP 2460 (Norcross, GA, USA) to obtain structural information about the material, including BET-specific surface area, pore size distribution, and pore volume. The material was degassed at 120 °C. X-ray photoelectron spectroscopy (XPS) measurements were carried out on an ESCALAB 250Xi (Thermo Scientic, Branchburg, NJ, USA) using monochromatized Al Kα radiation (1486.6 eV). The XPS spectra were energy calibrated by setting the adventitious carbon peak to 284.6 eV.

### 2.3. Electrochemical Test

#### 2.3.1. Three-Electrode System

The electrodes were prepared from ACs by adding polytetrafluoroethylene emulsion (PTFE) as a binder and acetylene black as a conductive agent with a NS-AC/acetylene black/PTFE mass ratio of 85/10/5 in ethanol to form the homogeneous slurry. Then, the slurry was evenly smeared on the Ni foam (1 cm × 1 cm) and dried in vacuum at 80 °C for 12 h, and then it was pressed with a tableting machine under 10 Mpa to obtain the working electrode. The mass loading of active materials on the working electrode was about 5.0 mg.

#### 2.3.2. Coin Cell Supercapacitor

The electrode was prepared in the same way as above, except that a circular electrode sheet (diameter of 8 mm) was used instead of a square electrode sheet. The symmetrical supercapacitor was constructed by using ACs as both the positive and negative electrodes. The electrolyte was a 6 M KOH aqueous solution on both sides of the separator (NKK TF4035). 

#### 2.3.3. Soft-Packaged Supercapacitor

The electrode was prepared using NS-AC-0.5-S20 by adding polytetrafluoroethylene emulsion (PTFE) as a binder and acetylene black as a conductive agent in ethanol, where the mass ratio of NAPAC/acetylene black/PTFE was 85/10/5. After rolling, compression, and drying at 120 °C for 12 h, the thickness of the electrode was controlled to be 0.5 mm. Then, the soft-packaged supercapacitor was built by packing two sheets of electrode and membrane in sequence and placing them into an Al plastic membrane. The organic electrolyte of 1 M TEATFB/PC (tetraethylammonium tetrafluoroborate/propene carbonate) was fed under atmospheric pressure in a glove box, where the oxygen and water content were less than 1 ppm. The soft-packaged supercapacitor was finally sealed and placed at room temperature for 24 h. The thin thickness of the device after compression is effective in minimizing the contact effect and obtaining low resistance.

#### 2.3.4. Electrochemical Measurements

Electrochemical measurements were carried out using an electrochemical workstation (CHI 760E, CH Instruments Inc., Shanghai, China). The potential window was −1.0 to 0 V in 6 M KOH for the three-electrode system at different scan rates. ACs on Ni foam, platinum foil, and Hg/HgO are the working electrode, counter electrode, and reference electrode, respectively. Cyclic voltammetry (CV) and galvanostatic charge–discharge (GCD) measurements were performed with a voltage window ranging from 0 to 1.0/1.5 V for the dual-electrode system and 0 to 2.5 V for the soft-packaged supercapacitor on a land cell tester (Landt, CT-2001A, Wuhan, China). Electrochemical parameters, including the specific capacitance (C, F g^−1^), energy density (E, Wh·kg^−1^), and power density (P, W·kg^−1^), were calculated according to the following equations:

(1) C=I×∆t/(m×∆V)

(2) $$E=C\times {∆V}^{2}/2$$

(3) P=E/∆t

where I (A) is the current for the GCD test, ∆t (s) is the discharge time, m (g) is the total weight of the sample in the Ni foam, and ∆V (V) is the corresponding potential window. All electrochemical measurements were carried out at room temperature without any atmospheric protection. To further investigate electrocatalytic kinetics, electrochemical impedance spectroscopy (EIS) measurements were carried out in the frequency range from 0.01 Hz to 100 kHz at an AC voltage of 5 mV.

The percent capacitive contribution at various scan rates can be calculated according to the following equation:

(4) $$i\left(V\right)=k_{1}v+k_{2}v^{1/2}$$

(5) $$C=\frac{1}{m\nu (V_{p}-V_{n})}\int_{V_{n}}^{V_{p}}i(V)dV$$

where i(V) is the current measured in a known potential V, ν is the scan rate with the unit of V s^−1^, and V_p_ and V_n_ are positive and negative potentials, respectively. Please note that the specific capacitance can be calculated using two methods: the GCD curve (Equation (1)) and the CV curves (Equation (5)).

## 3. Results and Discussion

### 3.1. Structure and Morphology of the ACs

**Figure 2:**
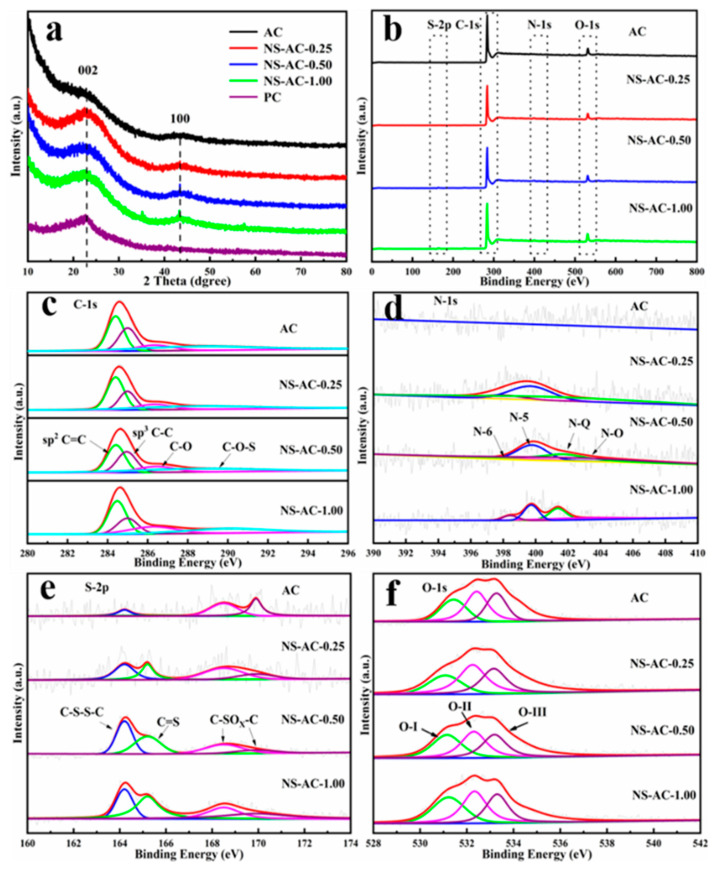
XRD pattern (**a**) and the XPS spectra (**b**) of the ACs and PC and fitted high-resolution XPS spectra of (**c**) C 1s, (**d**) N 1s, (**e**) S 2p, and (**f**) O 1s for ACs.

The XRD pattern of the ACs samples is shown in Figure 2a. PC is pristine carbon without activation. Two characteristic diffraction peaks at 2θ = 23.0° and 43.3° are assigned to the (002) and (100) reflections, typical for carbon materials [25,26,27]. The intensity of the first peak has a wide bandwidth, and the slope of the peak at a small angle from 10° to 15° is sharp, which suggests typical structural characteristics of amorphous porous carbon materials with rich nanoporous structures [28]. The wide bandwidth and weak (100) reflections indicate the degree of misalignment and low graphitization. As the amount of dye increased, the peak became sharp, indicating that the addition of dye played a positive role in increasing the degree of graphitization. The XRD result above confirms that the structure of petroleum coke precursor has been successfully transformed into a porous carbon structure by KOH activation at high temperatures.

The chemical composition of the AC samples was analyzed by XPS. The results are shown in Figure 2b. All the samples reveal a dominant C 1s peak at 284.6 eV, accompanied by slightly weak signals of N 1s, O 1s, and S 2p (Figure 2b), where the atomic ratios of C, N, O, and S are shown in Table 1, respectively. Since the content of the N and S elements is low relative to the high C content, the peaks are almost invisible and can be better seen from the N1s (Figure 2d) and S2p (Figure 2e) spectra. The C 1s spectra can be deconvolved into four peaks located at the binding energies of 284.6 eV, 285.1 eV, 286.5 eV, and 288.6 eV, corresponding to the sp_2_ C=C, sp_3_ C-C, C-O, and C-O-S, respectively [29,30,31]. Moreover, the N 1s XPS spectra revealed that ACs contain graphitic nitrogen (N-Q, 401.7 eV), pyridinic nitrogen (N-6, 398.4 eV), pyrrolic nitrogen (N-5, 399.7 eV), as well as N-O (403.0 eV) [32,33]. Notably, the concentrations of N-Q and N-5 doping are significantly higher than those of N-6 and N-O, which may be caused by the amino group in the dye wastewater. It is well known that N-6 and N-5 on the surface can provide pseudo-active sites, and graphitic nitrogen is good for conductivity. Thus, the addition of nitrogen dopant plays a positive role in the electrochemical performance of the electrode. The S2p XPS spectra shows four peaks at binding energies of 164.2 eV, 165.2 eV, 168.5 eV, and 169.9 eV (Figure 2e), corresponding to C–S–S–C, C=S, and C–SO_x_–C (x = 2 or 3) [34], respectively. Thus, it shows the successful incorporation of sulfur dopants into the carbon skeleton, which can also contribute to pseudocapacitance [35]. 

The O content is about 6–7 at.%, and the O 1s spectra can be deconvoluted into three characteristic peaks: C=O at 531.2 eV (O-Ⅰ), oxygen adsorbed on the surface (C-OH at 532.4 eV, O-Ⅱ), and adsorbed molecular water (-COOH at 533.4 eV, O-Ⅲ) [36]. Owing to their hydrophilic nature, these oxygen-containing functional groups can reduce the contact resistance of the surface of the electrode material with the electrolyte. The scale-up sample has the same results as the small-scale sample, indicating the feasibility of a large-scale industrial process. Based on XPS, N and S have been successfully doped into the C framework. The presence of N, S, and O can introduce defect sites and improve the wettability of the surface, which is beneficial for electrochemical processes.

**Figure 3:**
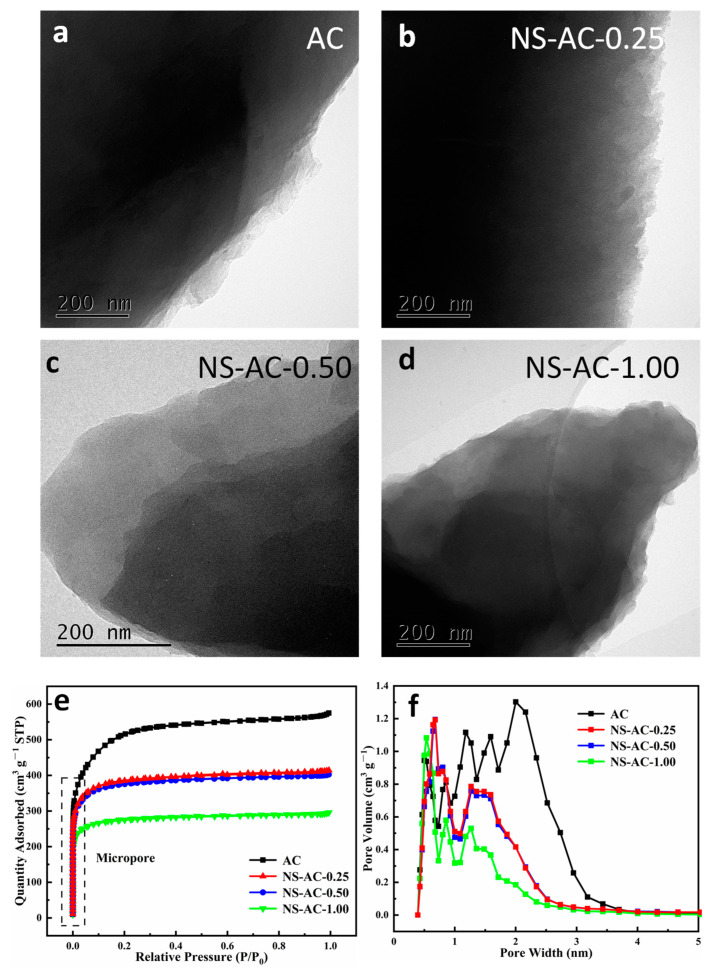
Typical TEM images (**a**–**d**) and the nitrogen adsorption/desorption analysis: (**e**) isotherm and (**f**) pore width distribution of the ACs.

The structure of ACs can be seen in Figure 3a–d. All samples showed a nanosheet morphology with a thinner layer; this 2D layer structure could provide more active sites, and a larger surface area enhances the electrode–electrolyte interface, promoting greater charge storage capacity. ACs exhibit a type I isotherm (Figure 3e), indicating a typical microporous structure [37,38]. The pore size distribution of the ACs by the N_2_ sorption method also reveals that the majority of the pore size is within the micropore range [39,40]. The BET-specific surface area of the sample is calculated to be 1445 m^2^ g^−1^, in which the micropore area is 1407 m^2^ g^−1^ and the external surface area is 38 m^2^ g^−1^. All samples have a pore size distribution in the range of 0.3–4 nm, indicating a majority of micropores with a small portion of mesopores. As the amount of dye increases, the pore size distribution shifts down to the submicropores, and the pore volume decreases relatively. When the amount is increased to 1.0 g, the micropores greater than 1 nm largely decrease, and the pore volume is reduced, as shown in Table 2. This implies that the addition of dye may inhibit large pore formation by partly blocking the pores. A suitable amount of dye can enhance the mesoporous structure, but if it exceeds a certain value, blocking of the porous structure will occur. Since the porous structure directly affects the performance of the electrode, a proper balance between the porous structure and the amount of dopant should be achieved. Rotenbergand [41] and co-workers and many other reports have shown the advantages of microporous carbon materials as electrode materials for supercapacitor. In fact, most commercial supercapacitors are based on microporous carbons. A large number of micropores (0.3–2 nm) with an interconnected open porous structure can shorten the diffusion pathway and provide an ‘ion highway’ during the adsorption and desorption processes, thus improving transfer efficiency [42].

**Figure 4:**
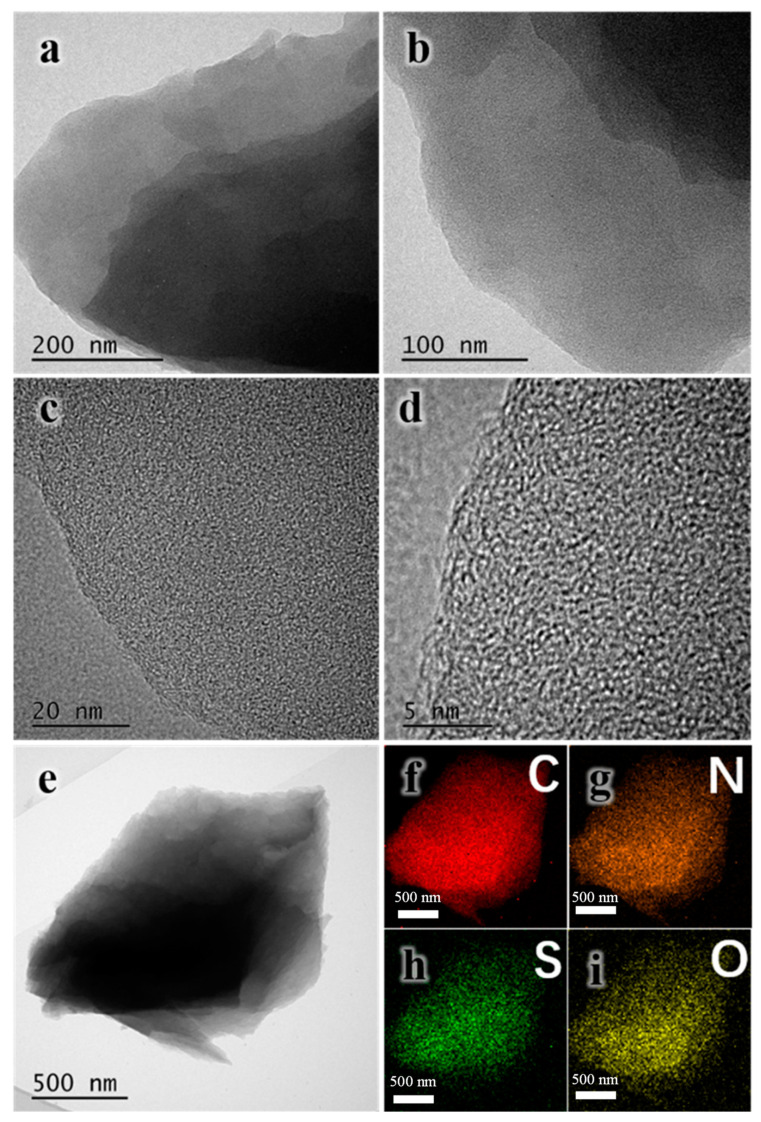
Typical HRTEM images (**a**–**d**) and the mapping (**e**–**i**) of NS-AC-0.50.

HRTEM images and the element distribution of C, N, S, and O in NS-AC-0.50 are shown in Figure 4. A more detailed sheet-like nanostructure can be seen in Figure 4a–d. The high-resolution image in Figure 4d shows a large number of defective twisted domains, showing very small imperceptible microporous structures (<2 nm). Elemental mapping (Figure 4f–i) clearly shows a uniform distribution of the elements C, N, O, and S within the sample.

In view of our synthetic process, the N and S from the dye could be transformed into a carbon skeleton and imbedded in the carbon framework at elevated temperatures. Part of the dye powder will decompose and constantly release abundant gases to generate porous structure during the carbonization process [43], and the generated gas will overcome the van der Waals force, increase the spacing of the graphite layer, and promote the doping of N and S into the carbon skeleton [27,44]. The generation of micropores is due to both KOH activation and the decomposition of the dye. 

### 3.2. Electrochemical Performances

**Figure 5:**
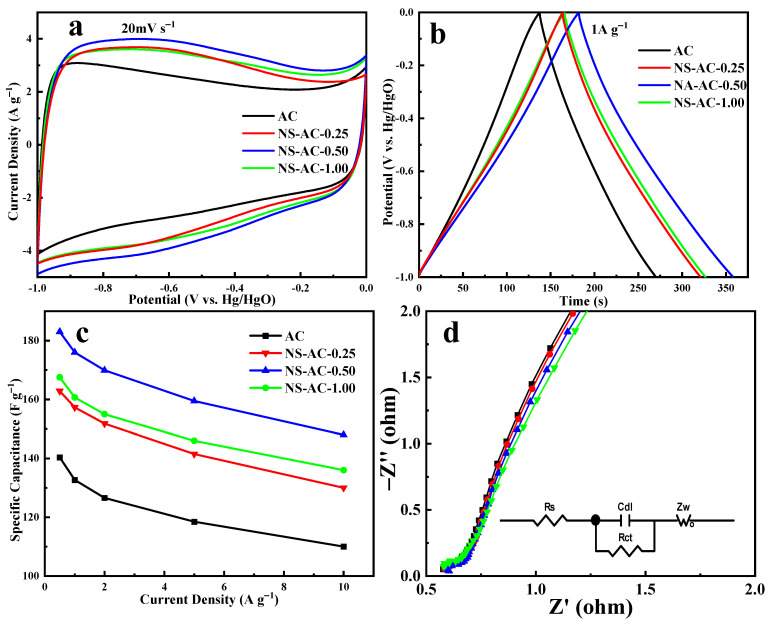
Capacitive performances of the ACs sample: (**a**) CV curves, (**b**) GCD curves, (**c**) rate capacitance curve, and (**d**) Nyquist plot. The inset is the equivalent electronic circuit used for EIS fitting.

The supercapacitor performances of the ACs material prepared from petroleum coke precursors have been studied in a 6 M KOH aqueous electrolyte. In the three-electrode test, NS-AC-0.50 has the best specific capacitance and rate performance (Figure 5a) in all samples due to its high specific surface area and the heteroatom doping. The CV curves at 20 mV s^−1^ show distorted rectangle shape, indicating the presence of both EDLC and pseudocapacitive characteristics. GCD curve in Figure 5b shows a relatively standard isosceles triangle and minimal undetectable voltage drop, suggesting low resistance and high charge efficiency. Figure 5c shows the rate performance of the sample at a current density of 0.5–10 A g^−1^. The specific capacitance of NS-AC-0.50 gradually decreases from 183 F g^−1^ at 0.5 A g^−1^ to 81% at 10 A g^−1^, suggesting comparative and better rate performance than AC. Electrochemical impedance spectroscopy (Figure 5d) further demonstrates that sample NS-AC-0.50 shows the lowest charge transfer resistance, and the insert is the equivalent electronic circuit used for EIS fitting [45]. The values of charge transfer resistance and solution resistance for all the electrodes after fitting are shown in Table 3. 

**Figure 6:**
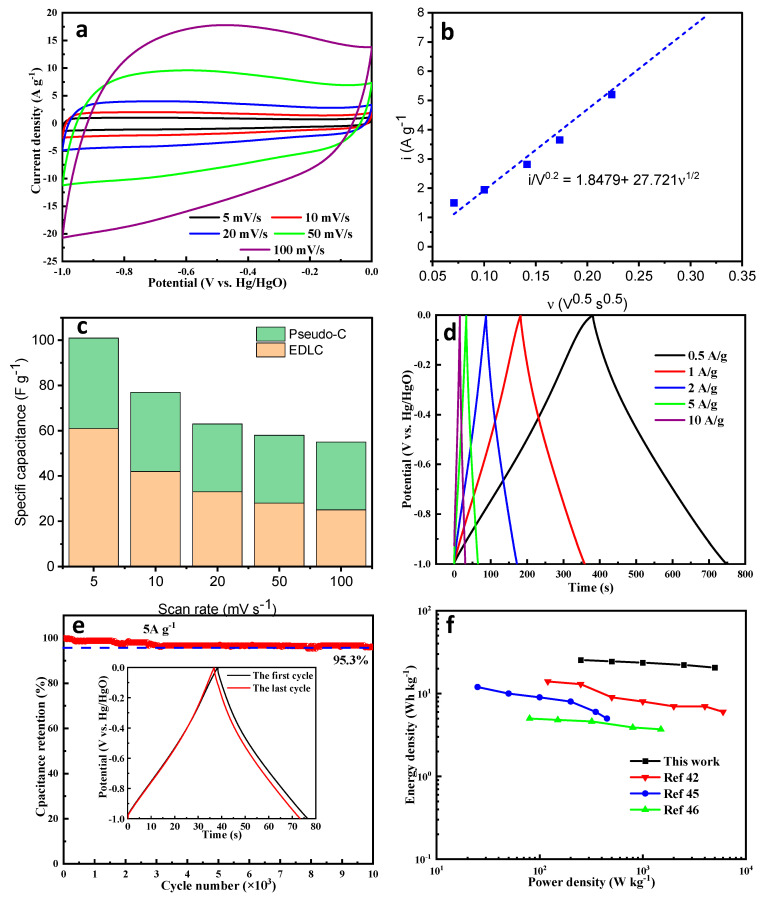
Capacitive performances of the NS-AC-0.50: (**a**) CV curves, (**b**) i(V) vs. ν^1/2^ plot under potential at 0.2 V vs. Hg/HgO, (**c**) the capacitance distributions of NA-AC-0.50 at different scan rates, (**d**) GCD curves, (**e**) Cycling performance of NA-AC-0.50 at 5 A·g^−1^ for 10,000 cycles (insert is the GCD curve of NS-AC-0.50 for the first and last cycles), and (**f**) Ragone plots showing the energy and power density of NS-AC-0.50 in comparison with the previously reported data.

NS-AC-0.50 was further evaluated due to its best performance among all the samples. As shown in Figure 6a,d, the shapes of the CV and GCD curves remain without much distortion with the increase in scan rate, suggesting good charge and discharge responses. Figure 6c,d summarizes the electrochemical capacitance of NS-AC-0.5 electrodes collected at different scan rates. Significantly, the NS-AC-0.5 electrode exhibited a good rate capability when the scan rate increased from 5 mV s^−1^ to 100 mV s^−1^, and a good EDLC distribution within the range of 50~60, suggesting the high performance of the electrode. The cycle stability of the material was tested at a current density of 5 A g^−1^, and the capacitance retention after 10,000 cycles was 95.3%, as shown in Figure 6e. The Ragone plot in Figure 6f shows an energy density of 24.4 Wh kg^−1^ at 500 W kg^−1^, outperforming some of the carbon-based materials previously reported [42,46,47]. These results indicate that the large specific surface area of the sample promotes the adsorption/desorption and transport of ions, and the rich porous structure promotes mass transfer, leading to superior rate performance and stability [48,49,50,51,52,53].

**Figure 7:**
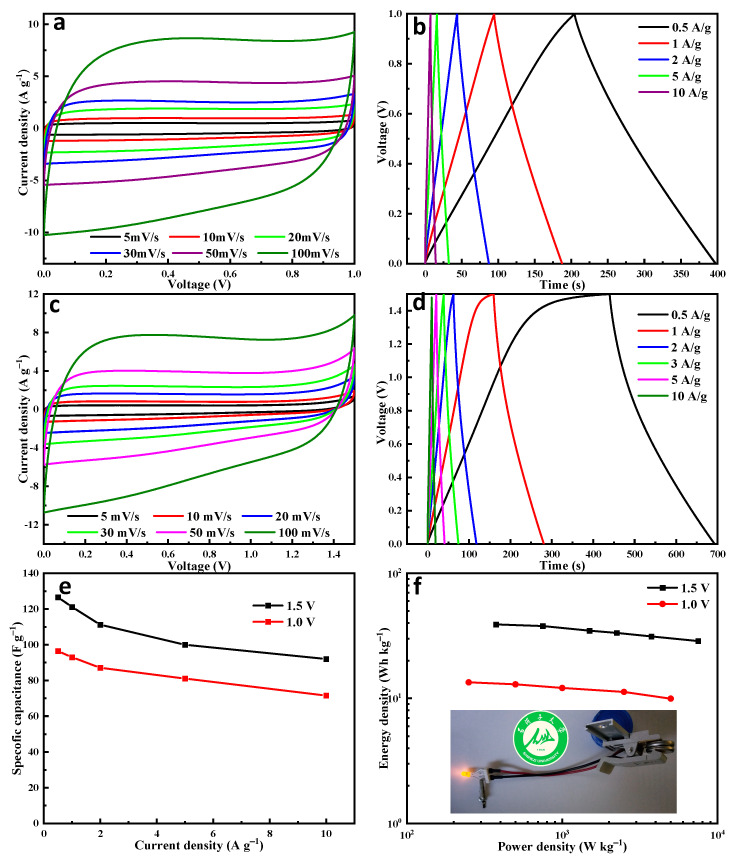
Capacitive performances of the NS-AC-0.50: (**a**) CV curves, (**b**) GCD curves with a window potential of 1.0 V, (**c**) CV curves with a window potential of 1.5 V, (**d**) GCD curves with a window potential of 1.5 V, (**e**) specific capacitance at potential windows of 1.0 V and 1.5 V, and (**f**) Ragone plots showing the energy and power density of NS-AC-0.50 in the two-electrode system with different window potentials (the insert is the image of the coin-cell supercapacitor).

We also constructed a symmetrical coin cell device based on NS-AC-0.50 as the positive and negative electrodes with a 6 M KOH electrolyte. The schematic diagram of the assembly is shown in Figure 7f. Good EDLC characteristics are seen in CV (Figure 7a) and GCD processes (Figure 7b). Figure 7e shows the rate performance of the device. Specific capacitance is 96.5 F g^−1^ at 0.5 A g^−1^ and can still remain as high as 71.5 F g^−1^ at 10 A g^−1^, indicating good rate performance. When the potential window increases to 1.5 V, the polarization phenomenon becomes more obvious at higher scan rates, as shown in Figure 7c. A specific capacitance of 121.1 F g^−1^ is obtained at 1 A g^−1^ with an operating potential window of 1.5 V (Figure 7d). Obviously, the energy density increases as the operating potential increases from 1 V to 1.5 V (Figure 7f). A high energy density of 37.8 Wh kg^−1^ at 750 W kg^−1^ is obtained when the potential window is 1.5 V.

### 3.3. Structure and Morphology of the Scaled Sample NS-AC-0.5-S20

**Figure 8:**
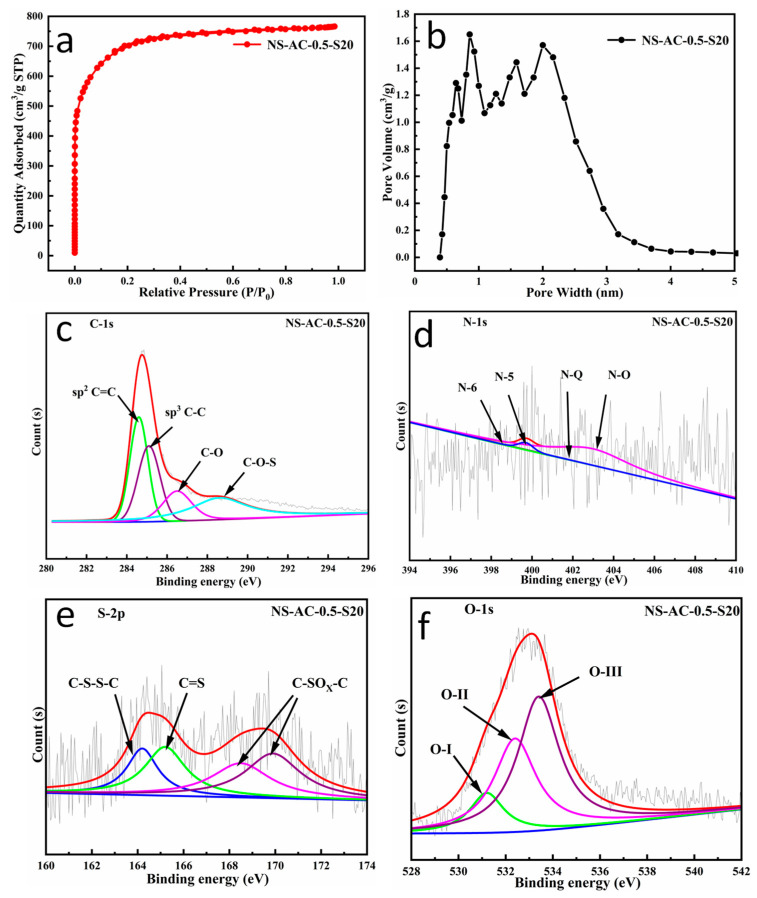
The nitrogen adsorption/desorption analysis: (**a**) isotherm and (**b**) pore width distribution and fitted high-resolution XPS spectra of (**c**) C 1s, (**d**) N 1s, (**e**) S 2p, and (**f**) O 1s for NS-AC-0.5-S20.

Figure 8a,b show the specific surface area and the pore size distribution of the scaled sample NS-AC-0.5-S20, which is produced at 20 kg/batch. A similar type I isotherm (Figure 8a) is obtained, which is consistent with that of the NS-AC-0.50. The BET-specific surface area of the sample is calculated to be 2582 m^2^ g^−1^, much higher than that of NS-AC-0.50. This is due to the superfine crushing in the scaled process, resulting in more efficient mixing and activation. The pore size distribution in Figure 8b indicates the majority of the pores are micropores (0.3 to 2 nm), similar to that of NS-AC-0.50. The pore volumes contributed by micropores and mesopores are 1.09 and 0.1 cm^3^ g^−1^, respectively. 

The XPS analysis results of the NS-AC-0.5-S20 sample are shown in Figure 8c–f. It reveals a dominant C 1s peak at 284.6 eV, accompanied by slightly weak signals of N 1s, O 1s, and S 2p, and the atomic ratios of C, N, O, and S are shown in Table 1, respectively. Similar information is obtained on the scaled sample except that the scale-up material shows higher concentrations of N-O and N-5 doping than N-6 and N-Q. The analysis of the S 2p XPS spectrum suggests four peaks at binding energies of 164.2 eV, 165.2 eV, 168.5 eV, and 169.9 eV (Figure 8e), and the O 1s shows similar four characteristic peaks as those of NS-AC-0.50. Moreover, the concentrations of C–SO_x_–C (168.5 eV, 169.9 eV, x = 2 or 3) [34], the adsorbed molecular water (-COOH at 533.4 eV, O-Ⅲ), and the O content are significantly higher than those of NS-AC-0.50, implying similar or better electrochemical performance might be obtained from the scaled product.

**Figure 9:**
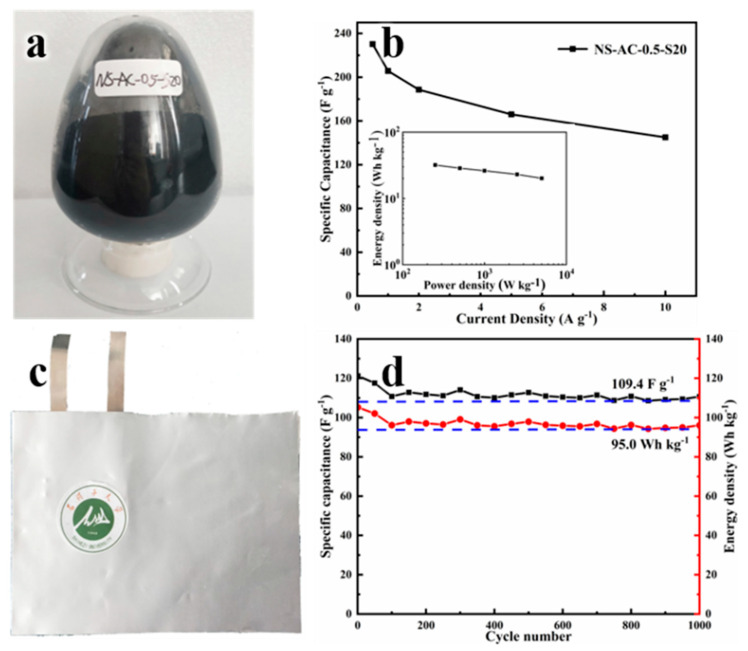
(**a**) The image of the NS-AC-0.5-S20 sample. (**b**) The rate capacitance curve of NS-AC-0.5-S20 in the three-electrodes system (the insert is the Ragone plots of the energy and power density of NS-AC-0.5-S20 in the three-electrodes system). (**c**) The image of the soft-package supercapacitor, the green image is the school badge of Shihezi University. (**d**) Cycling performance of NS-AC-0.5-S20 with 2.5 V.

As far as we know, very little literature research is done on material that is produced on a large scale [28]. Therefore, in order to show the practical industrial application, we have demonstrated both the large-scale production from the waste at 20 kg/batch and the full cell evaluation of the scaled sample. From Figure 9b, it can be seen that the scaled sample shows similar and even better electrochemical performance than the small-scaled sample in a three-electrode system with KOH as the electrolyte, owing to the higher specific surface area of the former. After packing our scaled sample into a soft-packaged supercapacitor cell (Figure 9c), its electrochemical performance was further tested in an organic electrolyte in the full symmetrical cell, which can be operated at a potential window of 2.5 V. Acetonitrile is commonly used in soft-packaged supercapacitors [12]. However, in view of its toxic and harmful nature, we use 1 M TEATFB/PC as the electrolyte. Our device exhibits a specific capacitance of 121 F g^−1^ at 0.2 A g^−1^ with 90% retention over 1000 cycles. This translates to an energy density of about 95 Wh kg^−1^ after 1000 cycles, which is much higher than that of commercial ACs and many small-scale functionalized carbons, highlighting its promising potential in both large-scale production as well as its applications in energy storage devices. 

## 4. Conclusions

In summary, we propose a novel synthetic strategy to prepare high-surface-area functionalized activated carbon on a large scale while simultaneously treating dye wastewater and upgrading petroleum coke. The N,S co-doped activated carbon produced in 20 kg/batch exhibited a high surface area of 2582 m^2^ g^−1^, a specific capacitance of 121 F g^−1^, and 90% capacitance retention at a potential window of 2.5 V after 1000 cycles in a soft-packaged full supercapacitor device. More importantly, these results demonstrate that dye wastewater is a promising modifier for carbon materials, and a new alternative and sustainable method to treat dye wastewater has been proposed. Solid waste and dye wastewater-derived activated carbon material is expected to be a promising ‘green’ energy storage material whose production provides a simultaneous solution for both environmental pollution and upcycling of industrial waste.

## Figures and Tables

**Table 1:** Chemical composition of ACs.

ACs	XPS (at.%)			
	C	N	O	S
AC	92.97	-	6.86	0.17
NS-AC-0.25	90.69	1.76	7.22	0.33
NS-AC-0.50	89.95	2.18	6.96	0.91
NS-AC-1.00	89.36	1.99	7.75	0.90
NS-AC-0.5-S20	90.39	1.60	7.46	0.54

**Table 2:** Pore parameters of ACs.

ACs	S_BET_, (m^2^ g^−1^)	Pore Volume, (cm^3^ · g^−1^)			V_mic_/V_t_, (%)	V_mes_/V_t_, (%)	D_ap_, (nm)
		V_t_	V_mic_	V_mes_			
AC	1903.0	0.89	0.81	0.08	91.0	9.00	1.87
NS-AC-0.25	1488.9	0.64	0.58	0.06	90.6	9.40	1.72
NS-AC-0.50	1445.3	0.63	0.57	0.06	90.5	9.50	1.72
NS-AC-1.00	1070.3	0.46	0.42	0.04	91.3	8.70	1.71
NS-AC-0.5-S20	2581.8	1.19	1.09	0.10	91.6	8.40	1.84

**Table 3:** Values of solution resistance and charge transfer resistance after EIS fitting.

Samples	Solution Resistance R_s_ (Ω)	Charge Transfer Resistance R_ct_ (Ω)
AC	9.56	0.26
NS-AC-0.25	9.56	0.25
NS-AC-0.50	9.56	0.23
NS-AC-1.0	9.58	0.26

## Data Availability

Data are contained within the article.
